# Different sea urchin RAG-like genes were domesticated to carry out different functions

**DOI:** 10.3389/fimmu.2022.1066510

**Published:** 2023-01-16

**Authors:** Iryna Yakovenko, Dror Tobi, Hadas Ner-Gaon, Matan Oren

**Affiliations:** ^1^ Department of Molecular Biology, Ariel University, Ariel, Israel; ^2^ Department of Computer Sciences, Ariel University, Ariel, Israel; ^3^ Department of Life Sciences, Ben-Gurion University of the Negev, Beer-Sheva, Israel

**Keywords:** RAG1, RAG2, sea urchin immunity, *P. lividus*, *S. purpuratus*, gene domestication, RAG transposon, RAG evolution

## Abstract

The closely linked recombination activating genes (*RAG1* and *RAG2*) in vertebrates encode the core of the RAG recombinase that mediates the V(D)J recombination of the immunoglobulin and T-cell receptor genes. *RAG1* and *RAG2* homologues (*RAG1L* and *RAG2L*) are present in multiple invertebrate phyla, including mollusks, nemerteans, cnidarians, and sea urchins. However, the function of the invertebrates’ RAGL proteins is yet unknown. The sea urchins contain multiple *RAGL* genes that presumably originated in a common ancestral transposon. In this study, we demonstrated that two different *RAG1L* genes in the sea urchin *Paracentrutus lividus* (*PlRAG1La* and *PlRAG1Lb*) lost their mobility and, along with *PlRAG2L*, were fully domesticated to carry out different functions. We found that the examined echinoid *RAGL* homologues have distinct expression profiles in early developmental stages and in adult tissues. Moreover, the predicted structure of the proteins suggests that while *PlRAG1La* could maintain its endonuclease activity and create a heterotetramer with *PlRAG2L*, the *PlRAG1Lb* adopted a different function that does not include an interaction with DNA nor a collaboration with *PlRAG2L*. By characterizing the different RAG homologues in the echinoid lineage, we hope to increase the knowledge about the evolution of these genes and shed light on their domestication processes.

## Introduction

The recombination activating genes, *RAG1* and *RAG2*, are the primary facilitators of the V(D)J recombination of the antibodies and T-cell receptor genes in the vertebrates (1, 2). The vertebrate RAG-1/2 complex recognizes and binds asymmetric recombination signal sequences (RSSs), flanking the V, D, and J gene segments of the *Immunoglobulin* (*Ig*) genes according to the 12/23 rule (3). The complex nicks the DNA helix at the junction with the coding gene segments. The intervening DNA is removed, and the randomly chosen coding segment pair is eventually ligated together (4). Just before the ligation of the segments, another diversification process occurs at the junction of the recombined genes segments. Several proteins are involved in the process, including general DNA non-homologous end-joining and repair enzymes and two dedicated V(D)J recombination proteins; the Artemis nuclease (also known as DNA Cross-Link Repair 1C – DCLRE1C) and the Terminal deoxynucleotidyl transferase (TdT). These proteins are involved in the nicking of the hairpin loop at the end of the pre-ligated coding segments and the addition of random N-nucleotides at the junction (5).

RAGs originated from a transposon but are thought to have been evolved predominantly in the vertical way (6–8). According to the transposon/split receptor gene hypothesis, the same transposon gave rise to vertebrate *RAG* genes and simultaneously caused the split of a primordial *Ig* gene that later resulted in the formation of the V, D and J segments (7, 9, 10). We recently proposed an alternative hypothesis in which a separate genetic element, not a *RAG1/2* transposon, was responsible for the *Ig* split. Based on the similarity between the RSSs and the Terminal Inverted Repeats (TIRs) of some *Transib* transposons, we suggested that the invading element which caused the *Ig* split was a *Transib*-like transposon (11). This hypothesis allows a more graduate, stepwise evolutionary explanation for the formation of the V(D)J recombination system in which the RAGs were first domesticated to carry out a different function and only later became a recombinase. We proposed that the initial role of the primordial vertebrate RAGs was to guard the vertebrate genome from the invasion of similarly structured harmful transposons.

*RAGs*-like (*RAGL*) genes are widely found in invertebrates, both in deuterostomes (echinoderms, cephalochordates and hemichordates) and in protostomes (mollusks, nemerteans and cnidarians). These invertebrate *RAGL* genes are found as *RAG1/2L* pairs, residing in transcriptionally convergent tail-to-tail configuration, as unpaired *RAG1L* genes and more rarely unpaired *RAG2L* genes, or as *RAGL* pseudogenes (12, 13). Many of the *RAGLs* still have some signatures of transposition activity, including Terminal Inverted Repeats (TIRs), the DNA recognition and cleavage sites, and 5 bp-long target site duplications (TSD) that are left on both DNA strands after a transposition (14, 15). A “living fossil” *RAG1/2L* transposon was discovered in lancelets and was termed *protoRAG* (16). This finding provided a proof of concept for the assumption the RAG1/2 loci (in both vertebrates and invertebrates) originated from a single transposon and suggested that active *RAGL* transposons may still exist. In fact, several such candidate transposons were suggested to exist in different taxonomic groups, such as insects (*N. geniculatus*), oysters and corals (13). On the other hand, some invertebrate *RAGL* genes lack TIRs and thus are suspected to be domesticated (13, 17).

In sea urchins, both *RAG1/2L* pairs and unpaired *RAG1L* genes were identified (10, 12, 13). Furthermore, it was demonstrated that two *Strongylocentrotus purpuratus RAG* genes, *SpRAG1* and *SpRAG2L*, are co-expressed in early developmental stages and in coelomocytes, and the encoded proteins create a stable complex, similar to the vertebrate RAG proteins (12). The *SpRAG1/2L* genes encode full-length proteins with all major functional domains as in the vertebrate RAGs, including a typical vertebrate RAG1 core with a DDE catalytic motif (17) and a functional PHD domain in SpRAG2 (18). Furthermore, SpRAG1L binds to the vertebrate RSSs with 12 bp spacers in the presence of vertebrate high mobility group (HMG) proteins (12).

Although we know that *RAG1L* and *RAG2L* genes in sea urchins are expressed, little is known about their specific expression patterns and location in early developmental stages and in adult tissues. In fact, the only verified information is from a single pioneer study about *RAG1L* and *RAG2L* genes in *S. purpuratus* (12). It is also unclear whether the transposition of *RAGs* is still ongoing in sea urchins. Furthermore, attention was so far paid exclusively to the *RAG1/2L* pair and not to the rest of the unpaired *RAGL* genes, and the functionality of these genes in sea urchins remains to be uncovered. In this study, we aimed to characterize and compare different *RAGL* gene sequences in sea urchins and to understand whether and when these genes were domesticated. We specifically chose to focus on two *P. lividus RAG1L* genes – the first (*PlRAG1a*) is in the same genetic locus (paired) with *PlRAG2L*, while the second (*PlRAG1b*) is unpaired. We found that the paired and unpaired genes are immobile and expressed differently in early developmental stages and in adults. Based on the above, we conclude that the two *PlRAGL* genes were recently domesticated to perform different, unrelated functions. An analysis of the functional protein structure suggests that PlRAG1a may work together with PlRAG2L as a nuclease while the PlRAG1b act alone and have adopted a different function altogether outside of the nucleus.

## Methods

### Culturing of sea urchins and obtaining embryos and larvae

Sea urchins (*P. lividus*) were obtained from the Israeli National Center for Mariculture in Eilat. Urchins were kept in artificial seawater (Red Sea Fish Pharm Ltd., Herzliya, Israel) in a 165-L aquarium in the Molecular Ecology Laboratory at Ariel University, of which 1/10 of the volume was replaced every week. The water salinity was kept around 40 ppt and the temperature between 18 and 22°C. The animals were fed once a week with either fresh or frozen *Ulva lactuca* algae. For obtaining *P. lividus* embryos and larvae, adult sea urchins were first injected with 0.5M KCl to induce spawning. Fertilization was made in filtered artificial seawater and developed embryos were maintained up to 72 hours post fertilization (pluteus larva, after which larvae start to feed).

### RNA isolation and cDNA creation

Total RNA was isolated with the RNeasy Mini Kit (Qiagen). The purification included a DNase treatment using the RNase-free DNase Set (Qiagen). The yield and purity and integrity of the RNA were measured using NP-80 spectrophotometer (Implen) and by gel electrophoresis. cDNA was created by qPCRBIO cDNA Synthesis Kits (PCR Biosystems, Tamar, Israel) according to manufacturer instructions.

### qPCR

Quantitative PCR reactions were executed in duplicates or triplicates in 96-well plates using QuantStudio real-time PCR (Thermo Fisher Scientific) with SYBR Green PCR mix (BioRad). Relative expression quantification was performed using delta-delta-CT with *PlTubulin* as a reference gene. To normalize the expression level across different tissues/organ, average expression value of the tested gene in each of the tissue/organ samples, was divided by the sum of the total expression of that gene in all tissues. Primers used: for *PLRAG1a (Pliv25095.1)* - F CCCTGGAAGAACTGGATGATAG R TCTTTGATGGTCGTCTCGATAAC, for *PLRAG1b (Pliv07077.1)* - F GAGGAGCTGGATGACCAAATTA R ACCATCTGCTCCATCCTTTATG, for *PLRAG2L (Pliv25741.1)* F GGAGATGCGATGACGGATATT R GATACGCCCTAGCTGTTGTT, for *PlArtemisL* (*Pliv23693.1*) - F CAACAGAGGGCAGTCTAACA R TGGTACTGGGTGTGATCATAAG, for *PlTdTL* (*Pliv29804.1*) - F CTAAGAACTCTGGGAGACGTAAAG R GGCGTGTTCAGTTCATCAAAG.

### *In-situ* probes preparation

*P. Lividus* RAG1b template sequence was amplified using the following primers – F AAACCAAAGGACGTCTCGCAAG R TGGAGTTACAGGGAGGCAGTCG. PCR amplicons were purified using QiaQuick PCR purification kit (Qiagen). Amplicons were then cloned into a pDrive cloning vector (Invitrogen) using the Qiagen PCR Cloning Kit (Qiagen) and transformed into *E. coli* bacteria. Positive colonies were grown in selective liquid LB media overnight, followed by purification using QIAprep Spin Miniprep Kit (Qiagen). The success of the cloning was verified by PCR and sequencing. DIG-labeled RNA probes were generated from the recombinant plasmids using DIG-RNA Labeling Kit (Roche). Sense and antisense probes were generated, using SP6 and T7 promoters flanking the cloning sites. Probes were stored in aliquotes at -80°C until use.

### Whole mount *In-situ* hybridization

The WhMISH was performed according to (19, 20). Briefly, the embryos were fixed with 4% formaldehyde, washed and prehybridized in the 96 well plates for 3 h in a hybridization buffer at 65°C. The hybridization was carried out overnight at 65°C. The embryos were thoroughly post-washed, blocked and incubated with AntiDIG-AP antibody overnight. The washed embryos were stained with NBT/BCIP and observed under a microscope.

### Databases and publications screening

The search for *RAGL* genes was performed in both annotated and not annotated sea urchin genomes by gene name and/or by sequence comparison using the SpRAG1L and SpRAG2L aa sequence. The comparisons were performed with tblastn (aa sequence against translated nucleotide sequences) against the NCBI gene bank and other databases of *S. purpuratus* (https://www.ncbi.nlm.nih.gov/assembly/GCF_000002235.5/)*, H. pulcherrimus* (https://cell-innovation.nig.ac.jp/cgi-bin/Hpul_public/Hpul_annot_home.cgi), *P. lividus* (https://www.ncbi.nlm.nih.gov/assembly/GCA_940671915.1/, http://octopus.obs-vlfr.fr/blast/oursin/blast_oursin.php), and *L. variegatus* (https://www.ncbi.nlm.nih.gov/assembly/GCF_018143015.1/). For *RAGL* expression data, a comprehensive search was performed in the following TSA databases: GHFM00000000.1, HAMP00000000.1, GHJZ00000000.1, GGVM00000000.1, GAPB00000000.1, GECD00000000.1, GAVR00000000.1, GAZP00000000.1, GAVF00000000.1, GCZS00000000.1, GEDS00000000.1, GFRN00000000.1, GIIR00000000.1, HACU00000000.1 corresponding to *S. purpuratus, Echinometra* sp.*, Mesocentrotus franciscanus, Loxechinus albus, Evechinus chloroticus, Arbacia punctulata, Echinarachnius parma, Eucidaris tribuloides, Sphaerechinus granularis* sea urchin species. Available literature was screened for *RAGLs* expression data in the sea urchins *S. purpuratus, Strongylocentrotus droebachiensis, L. variegatus, Psammechinus miliariswas*, results were organized as a table (**Table S4**).

### *RAG* expression analysis

To identify expression level we analyzed data from four publicly available RNA-seq datasets (GSE149221 – single cell experiment (21), GSE134350 – single cell experiment (22), GSE97448, PRJNA81157 (23, 24). Those datasets profiled developmental stages or immune challenges of *S. purpuratus* tissues. When the expression table was available, it was downloaded from the experiment site. In the single-cell experiments, python programs were written to sum up the expression for each gene in all cells. In the case of PRJNA81157, reads were downloaded and mapped to *S. purpuratus* genome (https://www.ncbi.nlm.nih.gov/assembly/GCF_000002235.5) using hisat2 (25) (version 2.0.5). Bam files were sorted and indexed by SAMtools (26) (version 1.9). The expression level (high, low or no expression) was determined by visually inspection using IGV tool (27). Low expression was regarded as less than 10 reads in the junctions.”

### Phylogenetic analysis

For the phylogenetic analysis we used sequences of which transcripts were identified with blast score > 100 and E value < 1e-18. Transcription information was obtained from TSA databases: GEDS00000000.1, GFRN01000000.1, GIIR00000000.1, HACU00000000.1 (*P. lividus*), GJVT00000000.1, GHFM00000000.1, GAVU00000000.1 (*S. purpuratus*), GAUR00000000.1 for *L. variegatus* and transcriptomic databases available in public genomic viewers for *H. pulcherrimus* https://cell-innovation.nig.ac.jp/cgi-bin/Hpul_public/Hpul_annot_home.cgi, and *P. lividus* https://www.ncbi.nlm.nih.gov/assembly/GCA_940671915.1/. Phylogenetic relationships of the echinoid RAG1L translated protein sequences were inferred with MUSCLE alignment with default parameters. Tested models included JTT and WAG, resulted in similar tree typologies. We chose to present a maximum likelihood method tree created with Whelan and Goldman (WAG) model (28) with a consensus tree inferred from 500 bootstrap replicates.

### Nanopore sequencing of amplified genomes from single sea urchin cells

Single sperm cells and coelomocytes of *S. pupuratus* were previously isolated using limited dilutions and subjected to whole genome amplification procedure (29). Amplified genome from one sperm and one coelomocyte were subjected to whole genome sequencing using MinION nanopore sequencing platform. The sequencing was performed according to Nanopore SQK-LSK109 protocol for long reads with MinION Nanopore apparatus (Oxford Nanopore Technologies). ~ 200 fmol of purified amplification products were subjected to DNA repair and end-prep using a NEBNext DNA repair mix and NEBNext Ultra II End Repair/dA-Tailing Module (New England Biolabs). DNA purification steps were carried out by the SPRI magnetic beads (Canvax, Spain). Base-calling was done automatically by the MinKnow program. Raw reads were obtained in FAST5 and FASTQ formats from which “pass” quality reads were cleaned from adaptor sequences by porechop (30) and uploaded to SRA database (accession SAMN31216822, SAMN31216823) and subjected to further analysis.

### Sequencing data analysis and assembly

Sperm and coelomocyte reads were sorted by quality by samtools (26) and aligned to the last version of *S. purpuratus* genome (Accession: GCF_000002235.5) by minimap2 (31), aligned reads were sorted and indexed by the samtools (26) and visualized in IGV genome browser (27). Long reads that overlapped with RAGs and/or the surrounding genes were identified by BLAST.

### Protein 3-D structure

The structure of the PIRAG1 type A and PIRAG2 complexes with DNA was modeled using th3 or comparative modeling software MODELLER (32). The template used is the structure of BbRAGL-3’TIR synaptic complex with nicked DNA (PDB code 6B40 (33)) from *Branchiostoma belcheri*. The percentage sequence identity (similarity) between sequence and it template structure is 42.4 (58.9%) % for PIRAG1 Type A and 30.6 (49.8) % for PIRAG2. At this level of sequence similarity, the resulting model is expected to show RMS error rises to about 1.5 Å for about 80% of residues from the correct structure as noted by Fiser A. Template-based protein structure modeling (34). The same nicked DNA of the template structure was used in the model. Inter chain (or subunits) residues with atoms within a cutoff distance of 4.5Å are defined as contacting residues (pyMOL).

## Results

### Echinoid *RAG1L* genes evolved through multiple lineages

**Figure 1:**
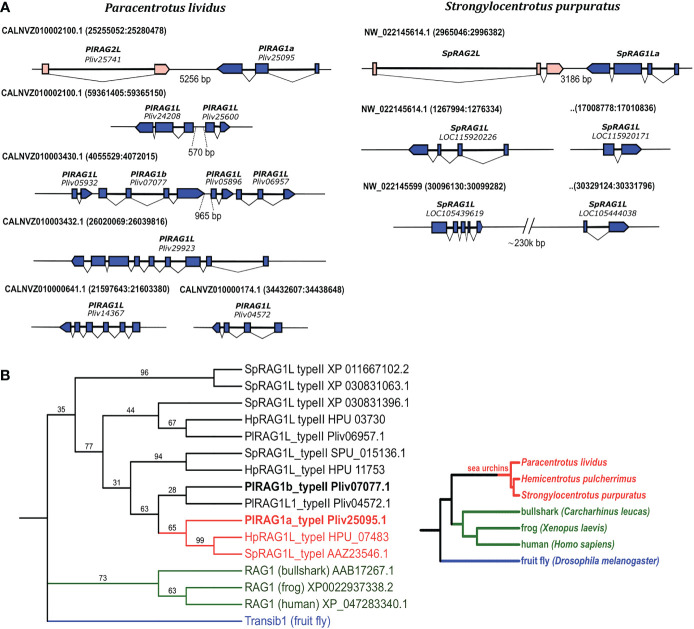
Genomic organization and phylogenetic relationships among echinoid RAG1L sequences. **(A)**. annotated *RAGL* genes in the genomes of *P. lividus* and *S. purpuratus*. **(B)**. phylogenetic relationships among echinoid RAG1L sequences. The phylogeny was made using transcribed sequences, based on maximum likelihood with Whelan and Goldman (WAG) model with consensus tree inferred from 500 bootstrap replicates. *Drosophila melanogaster* Transib autonomous protein was chosen as the outgroup (repbase https://www.girinst.org/server/RepBase/index.php accessed on July 2022). A corresponding guideline phylogeny of species is presented on the right to the phylogenetic relation of *RAG1L* translated protein sequences.

**Figure 2:**
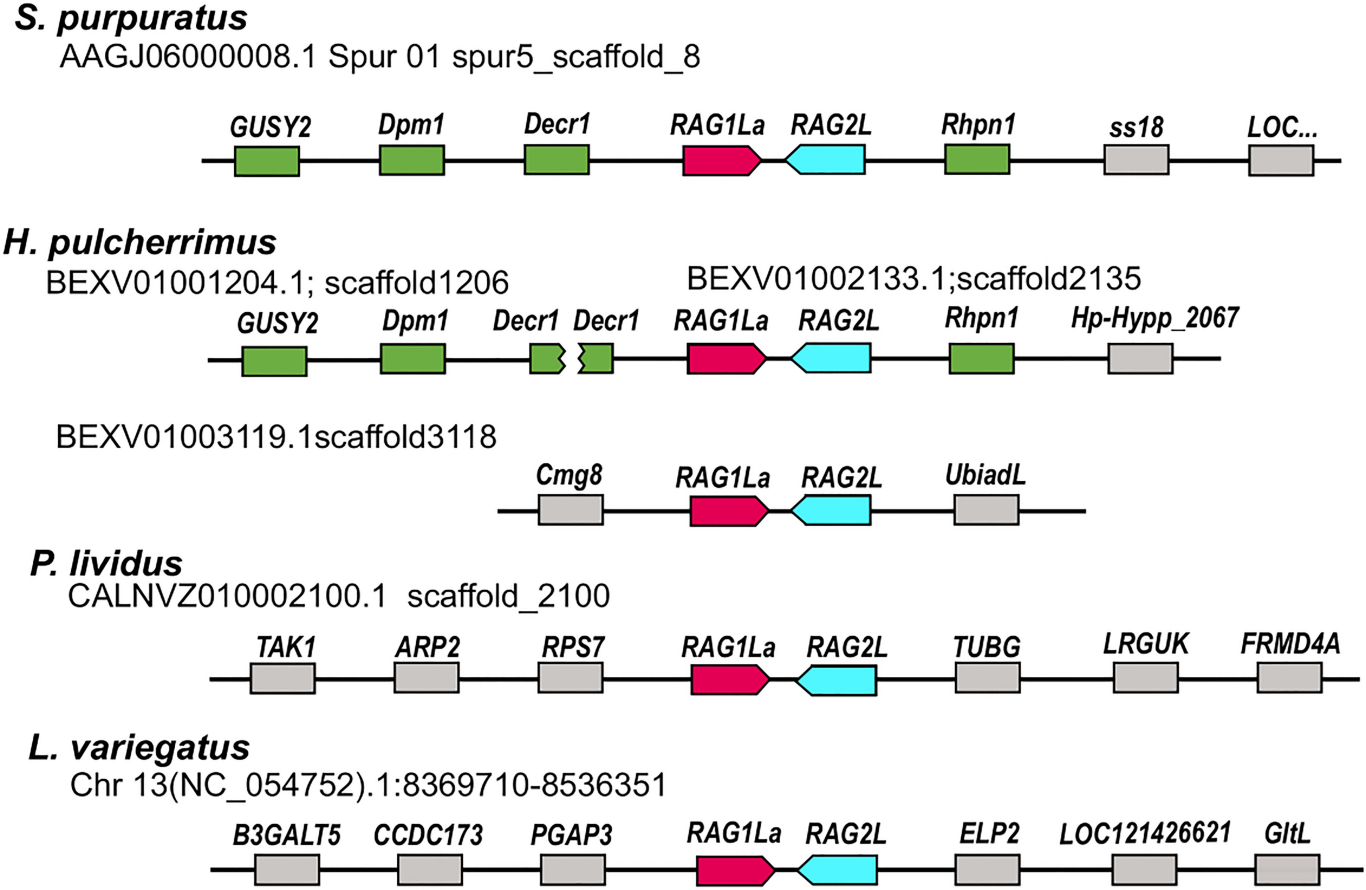
Relative genomic locations of type I *RAGL* genes in sea urchin genomes. The relative genomic location of *RAG1/2* genomic pairs in *S. purpuratus*, *P. lividus, H. pulcherrimus* and *L. variegatus* is presented. Matching neighboring genes are marked in green. The SpRAG1/2L and HpRAG1/2L loci are similarly neighboring rhodophilin-1 (*Rhpn1*) gene upstream to the RAG2L sequence. On the other side, upstream to the RAGL1 sequence, the RAG loci are adjacent to 2,4-Dienoyl-CoA Reductase-1 (*DECR1*) gene. The *H. pulcherrimus* genome scaffold BEXV01002133.1 is ending in the middle of *DECR1* gene but another scaffold - BEXV01001204.1 includes the rest of the *DECR1* gene sequence as well as two other genes with similarity to Dolichol-phosphate mannosyltransferase subunit 1 (*DPM1*) and Retinal Guanylate Cyclase 1-like (*GUSY1*) similarly to the corresponding neighboring *RAG1/2L* genes of the *S. pupuratus* genome.

To characterize the different sea urchin *RAG1L* genes, we investigated four available sea urchin genomes of *S. purpuratus*, *P. lividus, Hemicentrotus pulcherrimus* and *Lytechinus variegatus*. In contrast to jawed vertebrates, which have a single *RAG1* copy, available sea urchin genomes contain multiple copies of *RAG1L* sequences. The genomes that were screened contained between three sequence matches (in *L. variegatus* genome) and up to 7 sequences (in *P. lividus*) with a similarity blast score of more than 100 when compared with the complete *SpRAG1L* sequence (**Table S1**). About 60% of the *RAG1L* sequences were not annotated as genes in the genomic databases and did not appear in any of the expression databases. We, therefore, regarded these sequences as potential pseudogenes, perhaps reminiscence of past transposition activity, and excluded them from our analysis. The sea urchin *RAG1L* genes can be fitted into one of two main categories according to their genomic organization: the first category consists of *RAG1L* genes that are found next to *RAG2L* genes in tail-to-tail transcriptionally convergent orientation (**Figure 1A**), in similar to the lancelet *protoRAG* and the vertebrate *RAGs* loci (from now on will be termed type I). *RAG1L* type I genes were found in a single copy in each of the sea urchin genomes except for *H. pulcherrimus*, in which two *RAG1L/RAG2L* loci were identified on two separate genomic scaffolds (scaffold:2135 BEXV01002133.1 and scaffold:3118 BEXV01003119.1) (**Figure 2**). The second *RAG1L* category, consists of solitary *RAG1L* copies that are spread throughout the genome (**Table S2**), often in small clusters and sometimes as potential pseudogenes (will be termed type II). Both *RAG1L* gene types consist of multiple exons, as in the sea urchins *P. lividus* and *S. purpuratus*, suggesting they may be transcribed in several alternative isoforms (**Figure 1A**).

To understand the phylogenetic relationship among echinoid *RAG1L* genes from the sea urchin species investigated, a phylogenetic tree was constructed based on the *RAG1L* translated amino acid sequences. Only the sequences previously annotated as genes or expressed (found in corresponding transcriptomic databases) were used for the tree. In the phylogenetic analysis, all echinoid *RAG1L* type I genes, except for one (HpRAG1L_type I HPU_11753), were clustered in a single clade (**Figure 1B** in Red), implying their homology to each other. On the other hand, *RAG1L* type II sequences were clustered in a polyphyletic manner in four clades, of which two were composed of genes from different sea urchin species (**Figure 1B** in Black). These results suggest that some of the sequences might have originated in separate *RAG* lineages formed within a common echinoid ancestor/s before the speciation events of the examined species took place.

### Mobile *RAGL* sequences were domesticated in specific sea urchin species

The *RAGL* genes were previously identified in non-syntenic regions in the genomes of different sea urchin species (35, 36). To expand this analysis based on the newly added and improved genomic data, we compared the relative genomic location of the *RAGL* genes in all four available sea urchin genomes based on the identification of the neighboring genes. In almost all cases, *RAGL* genes (both type I and II) were found in non-synonymous positions in the echinoid genomes (**Figure 2**; **Table S2**). One exception was the *SpRAG1L* type I (accession: NW_022145614.1) and the *HpRAG1L* type I (located on scaffold BEXV01002133.1) that, together with their adjacent echinoid *RAG2L*, were found to be in corresponding genomic loci (**Figure 2** genes marked in green). These findings suggest that *RAG1L* type I immobilization in these two species already occurred in a common ancestor around 6 to 8 million years ago when the divergence of these species took place (37, 38).

**Figure 3:**
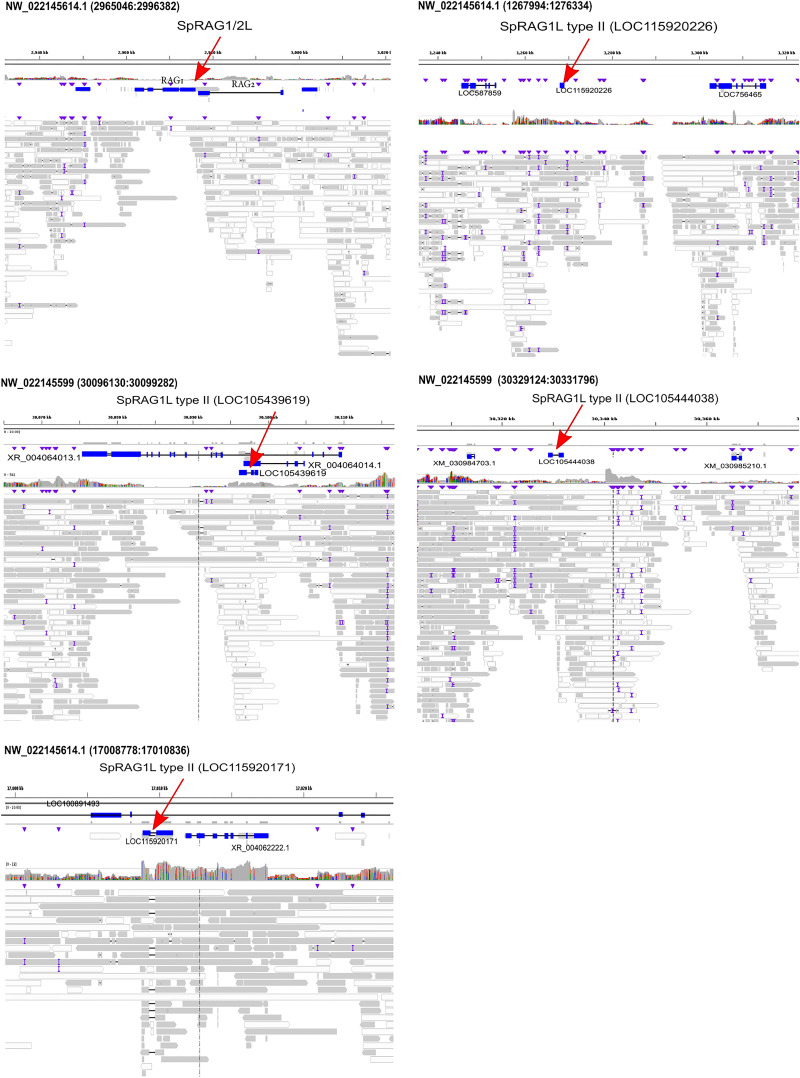
*SpRAGL* genes are immobile in individual sperm and a coelomocyte. The IGV visualized alignment is shown for *SpRAG1L* type I and II (*LOC115920226, LOC105439619, LOC105444038, LOC115920171*) genes and their neighboring genes. Red arrows pointing to the *RAG1* genes, purple triangles mark the mismatches. Reads with good mapping quality (over 60) are marked in grey, the reads with lower quality mapping are in white.

To test whether the sea urchin *RAG* genes are still mobile today, we performed a nanopore long-read sequencing of amplified genomes of a single sperm cell and a coelomocyte from *S. purpuratus* that were combined for the alignment of the reads to the *S. pupuratus* genome (GCF_000002235.5). The alignment suggested that all *RAG1L* sequences were not mobile and remained in synonymous positions in both *S. purpuratus* genomes. We note that because of the low coverage (3-5x), we were not able to assemble the genome fully. However, using the nanopore long reads, we were able to identify overlaps between the reads and large genomic areas upstream and downstream of the genes, which confirmed the linkage between the *RAG1L* genes and the neighboring genomic sequences and genes (**Figure 3**). We, therefore, considered the data reliable enough to conclude that the *RAG1L* genes are located within the same loci for this species and not active transposons.

### *PlRAG1a* and *PlRAG1b*: Similar genes with opposing expression patterns

To characterize the expression patterns of *P. lividus RAG1L* genes, we chose two transcribed genes (*P. lividus* database accessions: Pliv25095.1 and Pliv07077.1), with complete ORFs, representatives of type I and type II *RAG1L* and named them *PlRAG1a* and *PlRAG1b* accordingly (**Figure 1**). We tested the expression patterns of these genes using qPCR and whole-mount *in-situ* hybridization (WMISH) in *P. lividus* early life stages and adult tissues. We also tested the expression levels of the *PlRAG2L* gene and two of the vertebrate V(D)J recombination gene homologues; *PlArtemis* (*Pliv23693.1*) and *Pl Terminal deoxynucleotidyl transferase* (*TdT*) (*Pliv29804.1*). In the vertebrate lineage, these two genes work together with the RAG1/2 complex and therefore are expressed in various organs and co-expressed in the thymus (39). The translated *PlArtemisL* protein sequence showed 64% aa identity to the human protein (XP_047281606.1), and the *PlTdT* had 37,5% aa sequence identity compared to its human counterpart (NP_004079.3).

**Figure 4:**
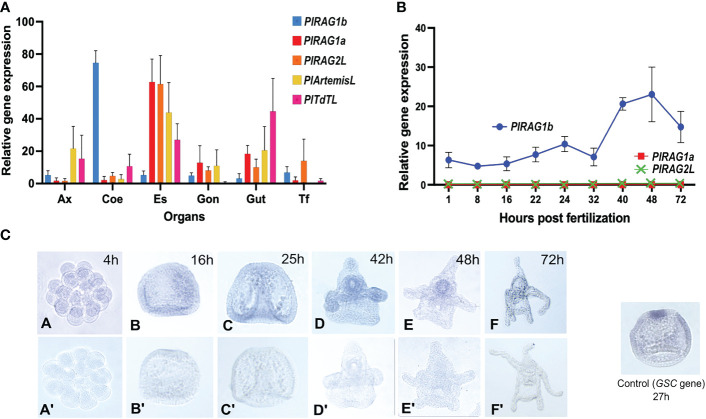
Expression patterns of *PlRAGL1a, PlRAGL2, PlRAGL1b*, *PlArtemisL* and *PlTdTL* genes in adult tissues and early developmental stages of *P. lividus* **(A)**. Relative gene expression in of the tested genes in adult sea urchin tissues and organs by qPCR. Ax - axial organ, Coe - coelomocytes, Es - esophagus, Gon - gonads, Gut, Tf - tubefeet. **(B)**. Relative gene expression of *PlRAG1a* and *PlRAG1b* in the early developmental stages by qPCR. The *PlRAG2L* expression is showed according to the PRJNA264358, PRJNA376650 and PRJEB10269 RNA-seq databases. **(C)**. *PlRAG1b* expression in the developmental stages visualized by the in-situ hybridization. Gene accession numbers in octopus database (http://octopus.obs-vlfr.fr/): *PlRAG1a* - Pliv25095.1 (type I), *PlRAG1b*- Pliv07077.1 (type II), *PlRAG2L*- Pliv25741.1, *PlArtemisL* - Pliv23693.1, *PlTdTL* - Pliv29804.1.

Our qPCR analysis showed the expression of all five tested genes in adult *P. lividus* tissue. Among them, the two *PlRAGLs* showed contrasting expression patterns. While *PlRAG1a* (type I) was co-expressed with *PlRAGL2* mainly in the upper digestive system – in the esophagus and to a lower extent in the gut and in the gonads, the *PlRAG1b* (type II) was almost exclusively expressed in coelomocytes. Interestingly, similar to *PlRAG1a* and *PlRAGL2*, *PlArtemis* and *PlTdT* were co-expressed in the esophagus and gut. However, not like *PlRAG1a* and *PlRAG2L*, they also showed a similar expression level in the axial organ (**Figure 4A**). To test the expression patterns of the two *P. lividus RAGs* in the early *P. lividus* developmental stages, we performed qPCR and WMISH using embryos and larvae from six post-fertilization time points - 4h, 16h, 25h, 42h, 48h, and 72h post fertilization (PF). The qPCR of the early life stages showed expression of *PlRAG1b*, peaking at the 48h pluteus larva while *PlRAG1a* and *PlRAG2* were not expressed at all (**Figure 4B**). The WMISH showed expression as early as the blastula stage where it was identified in the endoderm (16h PF), continuing through the pluteus larvae stages where it was found in the early gut (42h-72h PF) peaking at 42-48h PF (**Figure 4C**).

To sum up the available knowledge on the expression of different *RAGs*, we searched available sequence read archives (SRAs) of *S. purpuratus* and transcriptome shotgun assembly (TSA) datasets (**Table S3**) of in the sea urchins *S. purpuratus, Echinometra* sp.*, Mesocentrotus franciscanus, Loxechinus albus, Evechinus chloroticus, Arbacia punctulata, Echinarachnius parma, Eucidaris tribuloides, Sphaerechinus granularis*. We also screened the literature for relevant expression (**Tables S4**). The *S. purpuratus* SRA datasets analyses (**Table S3**) concluded in ambiguous results. Some developmental expression datasets, (e.g. GSE149221) clearly showed the presence of *SpRAG1L* and *SpRAG2L*, while others (e.g. GSE134350) resulted in non or very few *RAGL* transcripts. Unexpectedly, adult tissue transcriptomic datasets GSE97448 and PRJNA81157 also showed very little *RAGL* expression (**Table S3**). In most datasets in both developmental stages and adult organs the type II RAG1L genes were not present. While separately analyzing the available literature and associated TSA databases, when available, we were able to see the expression of *RAGL1* type I and *RAG2Ls* in early developmental stages in the sea urchins *S. purpuratus* and *Echinometra* sp. (**Table S4**). *RAG1L* type I was also expressed in adult tissues of the sea urchins *Psammechinus miliaris, Strongylocentrotus droebachiensis and L. variegatus*. In *P. lividus* developmental transcriptomic dataset (**Table S4**), RAG1L type II transcripts were present in all tested developmental stages while transcripts of PlRAG type I and PlRAGL2 were (with the exceptions of low expression in PRJNA264358 and PRJNA376650), not found. Overall, this data agrees with our expression data.

### Predicted *PlRAG1* protein structures suggest that they differ in their intercellular location and function

The modular core of jawed vertebrate RAG1 protein is made of the domains that help it to bind to the DNA, particularly to recognize a nonamer and heptamer parts of DNA signal sequences and perform its endonuclease activity. Traditionally, both RAG proteins are subdivided into a “functional protein core” (384-1008 of RAG1 and 1-387 of RAG2 in mouse) that was proven to be able to carry out recombination on its own (40) and an N-terminal “non-core” regions. The RAG1 core includes a nonamer-binding domain (NBD), dimerization and DNA-binding domain (DDBD), pre- and RNase H domains (preR and RNH), Zn-binding domains, and the C-terminal domain (CTD) (41–43). The RAG2 core includes a six-bladed beta-propeller (WD40 repeat) domain, which interacts with both RAG1 and the DNA of the coding segment next to the heptamer sequence (44). RAG1 N-terminal “non-core” region contains structurally undefined regions as well as a Zinc Dimerization Domain (ZDD) that plays a role in the dimerization, stability and fidelity of the recombinase, and within it, the RING Zn finger motif that plays a role in polyubiquitination of RAG leading to the enhanced recombination activity (45, 46, 47). Additionally, protein domain prediction tools (e.g. InterPro) also recognize the importin binding domain (Imp-bd) in the N-terminus of RAG1 (48). The non-core region of RAG2 consists of a zinc finger plant homeodomain (PHD), which binds to the N-terminal tail of methylated histone 3 (H3K4me3) and is required for RAG2 correct interaction with the open chromatin during recombination (49).

**Figure 5:**
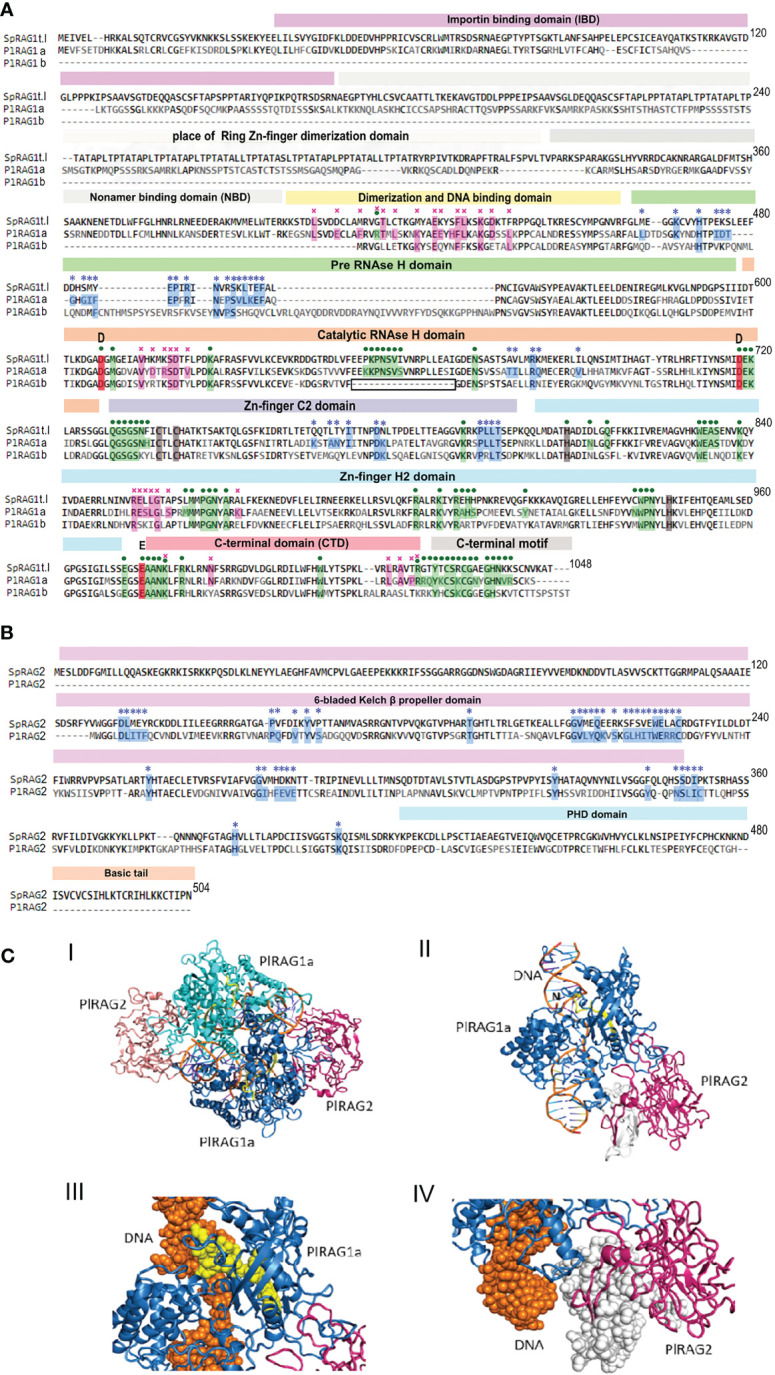
Predicted protein structure of PlRAGLs. **(A)**. sequences alignment of SpRAG1L, PlRAG1a and PlRAG1b with domains (Martin, Vicari et al., 2020). blue stars – contacting residues with RAG2 (RAG1 for RAG2), purple crosses - RAG1 dimerization contacts, green dots - contacting residues of RAG1 with DNA. Grey areas - places that are aligned to the ZDD and NBD domains of *Mus musculus* RAG1 with low similarity. Boxed section - missing sequence in the catalytic RNH domain in the RAG1Lb protein sequence. **(B)**. SpRAG2L and PlRAG2L sequence alighnment. **(C)**. Model structure of P. lividus Rag1L-Rag2L-nicked DNA complex. **(i)** The *P. lividus* Rag1/2L_DNA dimeric structure is shown using cartoon representation. One PlRag1L/PlRag2L complex is colored skyblue/hotpink and the other cyan/salmon, with the DNA colored orange. **(ii)** Monomeric half of the PlRAGL complex with the exposed DNA nick (marked with ‘N’). The region colored in yellow is missing in the PlRAG1b predicted protein. The region colored in white is missing in the PlRAG2L compared to SpRAG2L **(iii)** Magnification of the missing DNA binding (in RNAse H domain) region in PlRAG1b (shown in yellow spheres) and its interaction with the nicked DNA helix (shown in orange) (IV) Magnification of the missing region in PlRAG2L (white spheres) as compared with the SpRAG2L. The figure was prepared using the PyMol software 3.

The protein domain search in the echinoid RAGL-encoding loci was done according to the pre-defined domains listed in (13). As reported earlier, the ZDD was completely missing from all studied echinoid RAGLs (12, 33, 36). For type I RAGs - traces of NBD domain were found (17% and 13% for SpRAG1L and PLRAG1L to the *Mus musculus* RAG1), while all other domains were present with various (18-67%) levels of similarity (**Table 1**). The echinoid type II RAG sequences, on the other hand, lacked the non-core region and the NBD in most of the sequences, whereas the “core” domains were either defined or had insufficient similarity or were completely absent (**Figure 5**).

**Table 1:** Domains presence in the echinoid RAGL genes.

	IBD	ZDD	NBD	DDBD	Pre-RNH	RNH	ZnBD	ZnFH2	CTD
S. purpuratus									
SpRAG1 typeI	✓	✗	✓	✓	✓	✓	✓	✓	✓
SpRAG1L 1 type II				✓	✓	partial	✓	partial	✓
SpRAG1L 2 type II				✓	undet.	partial	✓	undet.	
SpRAG1L 3 type II				✓	✓	✓	✓	undet.	
P. lividus									
PlRAG1a	✓	✗	✓	✓	✓	✓	✓	✓	✓
PlRAG1b				✓	✓	✓	✓	✓	✓
PlRAG1 1 type II						partial	✓	undet.	
PlRAG1 2 type II						partial	✓	undet.	
H. pulcherrimus									
HpRAG1 type I	✓	✗	✓	✓	✓	✓	✓	✓	✓
HpRAG1 1 type I	✓	✗	✓	✓	✓	✓	✓	✓	✓
HpRAG1 2 type II				✓	✓	partial	✓	✗	

Multiple alignment of the translated protein sequences of the two *PlRAGs* and the type I *S. purpuratus* RAG1L (NW_022145614.1) showed high degree of sequence similarity among the proteins (**Figure 5A**). PlRAGL1a (type I) showed 95% of translated protein sequence coverage and 50% sequence identity compared to only 56% sequence coverage and 50% identity for PlRAGL1b to the SpRAG1l protein sequence. While PlRAGL1a seems to include all mentioned domains as in SpRAG1L, its N-terminal 1-159 aa sequence, which includes the ZDD, showed a lower degree of conservation (29% aa identity) (**Figure 5A**). On the other hand, the core catalytic domains and the C-terminal region were found to be highly conserved with 67% aa identity. Compared with the *S. purpuratus* RAG1L type I, the translated PlRAGL1b protein sequence lacks its first 407 aa (1–407) of the N-terminus non-core region, as well as an 18 aa-long section of the catalytic RNH-like domain (**Figure 5A**, boxed). On the other hand, it includes an additional 20 aa-long and 38 aa-long sequences in the middle of the RNH domain. The translated PlRAG2L protein sequence was found to be shorter than SpRAG2L, with 126 aa-long sequence missing from its N-terminal part. The rest of the protein sequence showed 50% identity to its *S. purpuratus* counterpart (**Figure 5B**).

A predicted 3-D model of the dimers complex of RAG1/2L_DNA and monomeric form in *P. lividus* (**Figure 5C** panels I and II, respectively) was build using homology modeling technique based on the crystal structure of the *Branchiostoma belcheri* protoRAG-DNA complex (50). Although missing non-core ZDD, our *P. lividus* model suggests the dimerization of a PlRAG1a is possible with the predicted 34 contacting residues and the formation of RAG1/2L complex with 37 contacting residues. The predicted RAG1/2L-DNA complex includes 72 contacting residues with the DNA strand (**Figure 5A**). We further used our *P. lividus* PlRAG1a- PlRAG2L-DNA model to compare it to the predicted structure of PlRAG1b and to SpRAG2L. In the first comparison, we found that the 18 aa-long missing “catalytic” section in PlRAG1b (**Figure 5A**, boxed) is a part that in PlRAG1a closely interacts with the area of the nick in the DNA double helix (**Figure 5C** panel III yellow). This section includes at least seven residues that could directly contact the DNA helix in the predicted RAG1/2L-DNA complex (**Figure 5A**, boxed section). We, therefore, assume that RAG1Lb, which lacks this section, has a weaker connection with the DNA and probably has no nuclease activity. The PlRAG2L seems to form a complex with PlRAG1a but is missing a 126 aa-long section of its N-terminal side of the core 6-bladed Kelch domain (**Figures 5B, C** panel IV white). Considering the fact that PlRAG1b has 20 fewer possible contacting residues with PlRAG2L, we suggest that PlRAG1b and PlRAG2L may not at all form a dimer.

## Discussion

The interest in the RAGs and their unique evolutionary histories have been growing ever since the discovery of the *RAG* genes in vertebrates. In recent years additional data has been accumulated about various *RAGL* genes in different taxonomic groups of invertebrates, portraying a much wider presence of these genes throughout the tree of life. With the absence of the V(D)J recombination targets in the vertebrate lineage and the structural differences, it is yet not clear what is the function of these genes in invertebrate animals. Previous studies revealed that sea urchin *RAGLs* didn’t contain TIR sequences (12, 13), suggesting that they may not be functional transposons. For over a decade the question was raised whether sea urchin *RAGLs* genes have been domesticated, but attention was usually paid to the paired *RAGL1/2* genes, because of their similar genomic organization to the vertebrate *RAG1/2*. On the other hand, unpaired *RAGL* genes were neglected as the potential pseudogenes and past transposon remnants. In this study, we categorized the available *RAGL* genes of sea urchins based on their genomic organization (paired vs. unpaired). We focused on *P. lividus* species, for which the *RAGL* genes were not yet characterized and demonstrate that two different *PlRAGL* genes, *PlRAGLa* and *PlRAGLb* are domesticated and may have different functions.

Although TIR sequences around *RAGL* genes were not identified in any of the echinoid genomes, the nonsynonymous positions of these genes in different sea urchin species indicate a previous transposition activity. To check whether the *RAGL* genes are still mobile, we conducted nanopore long-read sequencing of single *S. purpuratus* cells and mapping of the reads to the existing *S. purpuratus* genome. Using this method, we confirm that sequences of both types I and II *RAGL*s are currently immobile in *S. purpuratus*. Furthermore, the *RAG1L* type I genes in two closely related sea urchin species- *S. purpuratus* and *H. pulcherrimus* were found in corresponding genomic locations, indicating that they have been immobile for at least 6 million years. Based on the assumption that all echinoid *RAGL* genes were originated in a single ancestor *RAG1/2L* transposon, which evolved through vertical evolution (8, 13, 51), we assume that the echinoid *RAGL* gene repertoire was shaped through a combination of intra-species transposition and duplication events in ancestral sea urchin species. This notion is further supported by the tendency for clustering of the genes that includes partial sequences and pseudogenes. Another evolutionary process that probably took place in the echinoid lineage is the acquisition of introns into the different *RAGL* genes. The sea urchin *SpRAG1/2L* genes contain multiple exons (12), while most vertebrate *RAG* genes, except for teleost fishes (52), consist of single exons. Interestingly, the amphioxus *ProtoRAG* also contains multiple exons but with introns in different sites and different intron phases (16). The presence of introns is a condition for alternative splicing variants (53), as was previously predicted but not proven for *SpRAGL* genes (12) which could not be shown in this study, due to the low expression rates of the *RAGL* genes in the sea urchin transcriptomic datasets.

Our finding shows that the *PlRAG1a* is co-expressed with *PlRAG2L* as well as with other homologues of vertebrates V(D)J recombination genes (i.e. *PlArtemis* and *PlTdT*). We cannot rule out the possibility that the *PlRAG1* and *PlRAG2* are working together with *PlArtemis* and *PlTdT* and maybe other V(D)J homologues, as a nuclease/recombinase complex, which may even be involved in increasing immune receptor diversity as in the vertebrates. However, no direct proof for the existence of such a complex was provided in this work and therefore we can only speculate at this stage. The co-expression of these genes in the adult tissues was identified mainly in the upper digestive system (esophagus and gut), which is not considered a specialized immune system organ. On the other hand, the upper part of the digestive system is perhaps the region, which is the most exposed to the external environment and thus is an area where immune protection is most needed.

Unexpectedly, *PlRAG1a* showed a different expression pattern from its *S. purpuratus* type I *RAG1L* equivalent. The explanation for this may be found in the comparative analysis of the 3-D structure of the RAG1/2L protein-DNA heterotetramer, which included differences in comparison to *S. purpuratus*. The differences in the complex structure between the two species include minor differences in the RAG1L protein structure (mainly the N-terminal side), and a major difference in the protein structure of the predicted PlRAG2L, which is missing the first 126 aa from its N-terminus when compared with the SpRAG2L sequence.

The predicted protein sequence and structure of *PlRAG1a* and *PlRAG1b* genes include two major differences First, the *PlRAG1b* is missing its N-terminus 1-159 residues, including the Importin binding domain, which functions in the binding of proteins to the importin transporter, which, in turn, carries the relevant proteins from the cytoplasm into the nucleus (54). The absence of the Importin binding domain suggests that the *PlRAG1b* encoded protein may not be internalized into the nucleus. The complementary evidence for the above notion is the absence of an 18 aa-long segment in the NBD of PlRAG1b. The segment seems to be important for the PlRAG1a interaction and perhaps nicking of the DNA double helix. The absence of these segment increases the chance that the PlRAG1b does not possess any nuclease activity.

While the role of the vertebrate RAGs is strictly immunological, in sea urchins, it may have been domesticated for other purposes. Nevertheless, the evidence for SpRAG1/2 complex formation (17), together with our prediction for the PlRAG1/2L complex, suggests that type I RAG1L proteins in sea urchins may still work as nucleases with or without the help of their corresponding RAG2Ls. One possible immunological target for the echinoid type I RAGL complexes is the *Transformers* (*Trf*) gene family (55–57) that was shown to be subjected to somatic gene diversification in individual cells (29) and to be present and functional in several sea urchin species (58). Further studies are needed to reveal the genomic targets of the echinoid RAGL complexes and to elucidate the specific role and mechanism of action.

## Data availability statement

The datasets presented in this study can be found in online repositories. The names of the repository/repositories and accession number(s) can be found below: https://www.ncbi.nlm.nih.gov/, PRJNA888396.

## Author contributions

IY and MO contributed to the development of the study IY, MO, DT, HN-G did the experimental part. IY and MO wrote the article. All authors contributed to the article and approved the submitted version.

## References

[1] Tonegawa S. Somatic generation of antibody diversity. Nature (1983) 302(5909):575–81. doi: 10.1038/302575a0 6300689

[2] Oettinger MA Schatz DG Gorka C Baltimore D. RAG-1 and RAG-2, adjacent genes that synergistically activate V (D) J recombination. Science (1990) 248(4962):1517–23. doi: 10.1126/science.2360047 2360047

[3] van Gent DC Ramsden DA Gellert M. The RAG1 and RAG2 proteins establish the 12/23 rule in V (D) J recombination. Cell (1996) 85(1):107–13. doi: 10.1016/S0092-8674(00)81086-7 8620529

[4] Yanagi Y Yoshikai Y Leggett K Clark SP Aleksander I Mak TW. A human T cell-specific cDNA clone encodes a protein having extensive homology to immunoglobulin chains. Nature (1984) 308(5955):145–9. doi: 10.1038/308145a0 6336315

[5] Kallenbach S Doyen N d'Andon MF Rougeon F. Three lymphoid-specific factors account for all junctional diversity characteristic of somatic assembly of T-cell receptor and immunoglobulin genes. Proc Natl Acad Sci (1992) 89(7):2799–803. doi: 10.1073/pnas.89.7.2799 PMC48750 1557386

[6] Sakano H Hüppi K Heinrich G Tonegawa S. Sequences at the somatic recombination sites of immunoglobulin light-chain genes. Nature (1979) 280(5720):288. doi: 10.1038/280288a0 111144

[7] Thompson CB. New insights into V (D) J recombination and its role in the evolution of the immune system. Immunity (1995) 3(5):531–9. doi: 10.1016/1074-7613(95)90124-8 7584143

[8] Fugmann SD. The origins of the rag genes–from transposition to V (D) J recombination. In: Seminars in immunology. Elsevier (2010) 22(1):10–16. doi: 10.1016/j.smim.2009.11.004 PMC2823946 20004590

[9] Schatz DG. Antigen receptor genes and the evolution of a recombinase. In: Seminars in immunology. Elsevier (2004) 16(4):245–56. doi: 10.1016/j.smim.2004.08.004 15522623

[10] Carmona LM Schatz DG. New insights into the evolutionary origins of the recombination-activating gene proteins and V (D) J recombination. FEBS J (2017) 284(11):1590–605. doi: 10.1111/febs.13990 PMC5459667 27973733

[11] Yakovenko I Agronin J Smith LC Oren M. Guardian of the genome: An alternative RAG/Transib Co-evolution hypothesis for the origin of V (D) J recombination. Front Immunol (2021) 12. doi: 10.3389/fimmu.2021.709165 PMC8355894 34394111

[12] Fugmann SD Messier C Novack LA Cameron RA Rast JP. An ancient evolutionary origin of the Rag1/2 gene locus. Proc Natl Acad Sci (2006) 103(10):3728–33. doi: 10.1073/pnas.0509720103 PMC1450146 16505374

[13] Martin EC Vicari C Tsakou-Ngouafo L Pontarotti P Petrescu AJ Schatz DG. Identification of RAG-like transposons in protostomes suggests their ancient bilaterian origin. Mobile DNA (2020) 11:1–20. doi: 10.1186/s13100-020-00214-y 32399063 PMC7204232

[14] Agrawal A Eastman QM Schatz DG. Transposition mediated by RAG1 and RAG2 and its implications for the evolution of the immune system. Nature (1998) 394(6695):744. doi: 10.1038/29457 9723614

[15] Hiom K Melek M Gellert M. DNA Transposition by the RAG1 and RAG2 proteins: a possible source of oncogenic translocations. Cell (1998) 94(4):463–70. doi: 10.1016/S0092-8674(00)81587-1 9727489

[16] Huang S Tao X Yuan S Zhang Y Li P Beilinson HA. Discovery of an active RAG transposon illuminates the origins of V (D) J recombination. Cell (2016) 166(1):102–14. doi: 10.1016/j.cell.2016.05.032 PMC5017859 27293192

[17] Carmona LM Fugmann SD Schatz DG. Collaboration of RAG2 with RAG1-like proteins during the evolution of V (D) J recombination. Genes Dev (2016) 30(8):909–17. doi: 10.1101/gad.278432.116 PMC4840297 27056670

[18] Wilson DR Norton DD Fugmann SD. The PHD domain of the sea urchin RAG2 homolog, SpRAG2L, recognizes dimethylated lysine 4 in histone H3 tails. Dev Comp Immunol (2008) 32(10):1221–30. doi: 10.1016/j.dci.2008.03.012 PMC2518978 18499250

[19] Ben-Tabou de-Leon S Su Y-H Lin K-T Li E Davidson EH. Gene regulatory control in the sea urchin aboral ectoderm: Spatial initiation, signaling inputs, and cell fate lockdown. Dev Biol (2013) 374(1):245–54. doi: 10.1016/j.ydbio.2012.11.013 PMC3548969 23211652

[20] Minokawa T Rast JP Arenas-Mena C Franco CB Davidson EH. Expression patterns of four different regulatory genes that function during sea urchin development. Gene Expression Patterns (2004) 4(4):449–56. doi: 10.1016/j.modgep.2004.01.009 15183312

[21] Foster S Oulhen N Wessel G. A single cell RNA sequencing resource for early sea urchin development. Development (2020) 147(17). doi: 10.1242/dev.191528 PMC7502599 32816969

[22] Foster S Teo YV Neretti N Oulhen N Wessel GM. Single cell RNA-seq in the sea urchin embryo show marked cell-type specificity in the Delta/Notch pathway. Mol Reprod Dev (2019) 86(8):931–4. doi: 10.1002/mrd.23181 PMC6690749 31199038

[23] Tu Q Cameron RA Davidson EH. Quantitative developmental transcriptomes of the sea urchin strongylocentrotus purpuratus. Dev Biol (2014) 385(2):160–7. doi: 10.1016/j.ydbio.2013.11.019 PMC3898891 24291147

[24] Tu Q Cameron RA Worley KC Gibbs RA Davidson EH. Gene structure in the sea urchin strongylocentrotus purpuratus based on transcriptome analysis. Genome Res (2012) 22(10):2079–87. doi: 10.1101/gr.139170.112 PMC3460201 22709795

[25] Kim D Paggi JM Park C Bennett C Salzberg SL. Graph-based genome alignment and genotyping with HISAT2 and HISAT-genotype. Nat Biotechnol (2019) 37(8):907–15. doi: 10.1038/s41587-019-0201-4 PMC7605509 31375807

[26] Danecek P Bonfield JK Liddle J Marshall J Ohan V Pollard MO. Twelve years of SAMtools and BCFtools. GigaScience (2021) 10(2). doi: 10.1093/gigascience/giab008 PMC7931819 33590861

[27] Thorvaldsdóttir H Robinson JT Mesirov JP. Integrative genomics viewer (IGV): high-performance genomics data visualization and exploration. Briefings Bioinf (2012) 14(2):178–92. doi: 10.1093/bib/bbs017 PMC3603213 22517427

[28] Whelan S Goldman N. A general empirical model of protein evolution derived from multiple protein families using a maximum-likelihood approach. Mol Biol Evol (2001) 18(5):691–9. doi: 10.1093/oxfordjournals.molbev.a003851 11319253

[29] Oren M Rosental B Hawley TS Kim G-Y Agronin J Reynolds CR. Individual sea urchin coelomocytes undergo somatic immune gene diversification. Front Immunol (2019) 10:1298. doi: 10.3389/fimmu.2019.01298 31244844 PMC6563789

[30] Wick RR Judd LM Gorrie CL Holt KE. Completing bacterial genome assemblies with multiplex MinION sequencing. Microbial Genomics (2017) 3(10). doi: 10.1099/mgen.0.000132 PMC5695209 29177090

[31] Li H. Minimap2: pairwise alignment for nucleotide sequences. Bioinformatics (2018) 34(18):3094–100. doi: 10.1093/bioinformatics/bty191 PMC6137996 29750242

[32] Sali A Blundell TL. Comparative protein modelling by satisfaction of spatial restraints. J Mol Biol (1993) 234(3):779–815. doi: 10.1006/jmbi.1993.1626 8254673

[33] Poole JRM Huang SF Xu A Bayet J Pontarotti P. The RAG transposon is active through the deuterostome evolution and domesticated in jawed vertebrates. Immunogenetics (2017) 69(6):391–400. doi: 10.1007/s00251-017-0979-5 28451741

[34] Fiser A. Template-based protein structure modeling. Methods Mol Biol (2010) 673:73–94. doi: 10.1007/978-1-60761-842-3_6 20835794 PMC4108304

[35] Kapitonov VV Jurka J. RAG1 core and V (D) J recombination signal sequences were derived from transib transposons. PloS Biol (2005) 3(6):e181. doi: 10.1371/journal.pbio.0030181 15898832 PMC1131882

[36] Kapitonov VV Koonin EV. Evolution of the RAG1-RAG2 locus: both proteins came from the same transposon. Biol Direct (2015) 10(1):20. doi: 10.1186/s13062-015-0055-8 25928409 PMC4411706

[37] Lee Y-H. Molecular phylogenies and divergence times of Sea urchin species of strongylocentrotidae, echinoida. Mol Biol Evol (2003) 20(8):1211–21. doi: 10.1093/molbev/msg125 12777524

[38] Kumar S Suleski M Craig JM Kasprowicz AE Sanderford M Li M. TimeTree 5: An expanded resource for species divergence times. Mol Biol Evol (2022) 39(8). doi: 10.1093/molbev/msac174 PMC9400175 35932227

[39] Maezawa S Nakano S Kuniya T Koiwai O Koiwai K. Double-strand break repair based on short-homology regions is suppressed under terminal deoxynucleotidyltransferase expression, as revealed by a novel vector system for analysing DNA repair by nonhomologous end joining. FEBS Open Bio (2016) 6(1):16–23. doi: 10.1002/2211-5463.12001 PMC4794791 27047738

[40] Kim DR Dai Y Mundy CL Yang W Oettinger MA. Mutations of acidic residues in RAG1 define the active site of the V(D)J recombinase. Genes Dev (1999) 13(23):3070–80. doi: 10.1101/gad.13.23.3070 PMC317176 10601033

[41] Gwyn LM Peak MM De P Rahman NS Rodgers KK. A zinc site in the c-terminal domain of RAG1 is essential for DNA cleavage activity. J Mol Biol (2009) 390(5):863–78. doi: 10.1016/j.jmb.2009.05.076 PMC2782368 19500590

[42] Kim MS Lapkouski M Yang W Gellert M. Crystal structure of the V(D)J recombinase RAG1-RAG2. Nature (2015) 518(7540):507–11. doi: 10.1038/nature14174 PMC4342785 25707801

[43] Ru H Chambers MG Fu TM Tong AB Liao M Wu H. Molecular mechanism of V(D)J recombination from synaptic RAG1-RAG2 complex structures. Cell (2015) 163(5):1138–52. doi: 10.1016/j.cell.2015.10.055 PMC4690471 26548953

[44] Callebaut I Mornon J-P. The V (D) J recombination activating protein RAG2 consists of a six-bladed propeller and a PHD fingerlike domain, as revealed by sequence analysis. Cell Mol Life Sci CMLS (1998) 54(8):880–91. doi: 10.1007/s000180050216 PMC11147292 9760994

[45] Rodgers KK Bu Z Fleming KG Schatz DG Engelman DM Coleman JE. A zinc-binding domain involved in the dimerization of RAG1. J Mol Biol (1996) 260(1):70–84. doi: 10.1006/jmbi.1996.0382 8676393

[46] Arbuckle JL Rahman NS Zhao S Rodgers W Rodgers KK. Elucidating the domain architecture and functions of non-core RAG1: The capacity of a non-core zinc-binding domain to function in nuclear import and nucleic acid binding. BMC Biochem (2011) 12(1):23. doi: 10.1186/1471-2091-12-23 21599978 PMC3124419

[47] Jones JM Gellert M. Autoubiquitylation of the V(D)J recombinase protein RAG1. Proc Natl Acad Sci U S A (2003) 100(26):15446–51. doi: 10.1073/pnas.2637012100 PMC307587 14671314

[48] Cortes P Ye ZS Baltimore D. RAG-1 interacts with the repeated amino acid motif of the human homologue of the yeast protein SRP1. Proc Natl Acad Sci U S A (1994) 91(16):7633–7. doi: 10.1073/pnas.91.16.7633 PMC44456 8052633

[49] Matthews AG Kuo AJ Ramón-Maiques S Han S Champagne KS Ivanov D. RAG2 PHD finger couples histone H3 lysine 4 trimethylation with V (D) J recombination. Nature (2007) 450(7172):1106–10. doi: 10.1038/nature06431 PMC2988437 18033247

[50] Zhang Y Cheng TC Huang G Lu Q Surleac MD Mandell JD. Transposon molecular domestication and the evolution of the RAG recombinase. Nature (2019) 569(7754):79. doi: 10.1038/s41586-019-1093-7 30971819 PMC6494689

[51] Flajnik MF Kasahara M. Origin and evolution of the adaptive immune system: genetic events and selective pressures. Nat Rev Genet (2010) 11(1):47–59. doi: 10.1038/nrg2703 19997068 PMC3805090

[52] Wang X Tan X Zhang PJ Zhang Y Xu P. Recombination-activating gene 1 and 2 (RAG1 and RAG2) in flounder (Paralichthys olivaceus). J Biosci (2014) 39(5):849–58. doi: 10.1007/s12038-014-9469-1 25431413

[53] Avgan N Wang JI Fernandez-Chamorro J Weatheritt RJ. Multilayered control of exon acquisition permits the emergence of novel forms of regulatory control. Genome Biol (2019) 20(1):1–13. doi: 10.1186/s13059-019-1757-5 31315652 PMC6637531

[54] Lott K Cingolani G. The importin β binding domain as a master regulator of nucleocytoplasmic transport. Biochim Biophys Acta (BBA) - Mol Cell Res (2011) 1813(9):1578–92. doi: 10.1016/j.bbamcr.2010.10.012 PMC3037977 21029753

[55] Nair SV Del Valle H Gross PS Terwilliger DP Smith LC. Macroarray analysis of coelomocyte gene expression in response to LPS in the sea urchin. identification of unexpected immune diversity in an invertebrate. Physiol Genomics (2005) 22(1):33–47. doi: 10.1152/physiolgenomics.00052.2005 15827237

[56] Terwilliger DP Buckley KM Brockton V Ritter NJ Smith LC. Distinctive expression patterns of 185/333 genes in the purple sea urchin, strongylocentrotus purpuratus: an unexpectedly diverse family of transcripts in response to LPS, beta-1,3-glucan, and dsRNA. BMC Mol Biol (2007) 8:16. doi: 10.1186/1471-2199-8-16 17331248 PMC1831783

[57] Oren M Hudgell MAB D’Allura B Agronin J Gross A Podini D. Short tandem repeats, segmental duplications, gene deletion, and genomic instability in a rapidly diversified immune gene family. BMC Genomics (2016) 17(1):1–19. doi: 10.1186/s12864-016-3241-x 27829352 PMC5103432

[58] Yakovenko I Donnyo A Ioscovich O Rosental B Oren M. The diverse transformer (Trf) protein family in the Sea urchin paracentrotus lividus acts through a collaboration between cellular and humoral immune effector arms. Int J Mol Sci (2021) 22(13):6639. doi: 10.3390/ijms22136639 34206148 PMC8268236

