# Supplementary material for: Different sea urchin RAG-like genes were domesticated to carry out different functions
Source: Front Immunol. 2023 Jan 16;13:1066510. doi: 10.3389/fimmu.2022.1066510 (PMC9885083; doi:10.3389/fimmu.2022.1066510)
Supplement: Supplementary file 1 [file Table_1.docx]

**Table S1. RAGL copy presence in the genomes of sea urchins***

| **Sea urchin** | **Database** | **Number of *RAG1L*** | **Number of *RAG2L*** |
| --- | --- | --- | --- |
| *Strongylocentrotus purpuratus* | AAGJ06 | 5 | 3 |
| *Paracentrotus lividus* | CALNVZ01 | 7 | 1 |
| *Heliocidaris erythrogramma* | JAMBMX01 | 5 | 1 |
| [*Hemicentrotus pulcherrimus*](https://www.ncbi.nlm.nih.gov/genome/24586) | BEXV01 | 4 | 2 |
| *Heliocidaris tuberculata* | JALRNC01 | 3 | 1 |
| *[Lytechinus pictus](https://blast.ncbi.nlm.nih.gov/Blast.cgi#alnHdr_1930237180)* | JADFUK01 | 5 | 1 |
| *Lytechinus variegatus* | JAFCXN01 | 3 | 1 |

* sequences with similarity blast score of more than 100 to *SpRAG1* protein sequence (GenBank accession no. DQ082723.1)

**Table S2. Neighboring genes of RAG1L type II (solitary)**

| **Sea urchin** | **Gene name** | **Upstream genes** | **Downstream genes** |
| --- | --- | --- | --- |
| ***S. purpuratus*** | ***  *LOC105439619*  *LOC105444038* | Unknown *LOC100889000,* ***ALP1***  *LOC100889399*, unknown *LOC115923624* | *LOC100889000, RAG1, LOC105444038, unknown*  *LOC100891548, LOC105444033, LOC100893037* |
|  | *LOC115920171* | overlapped by ***MAOB*** *LOC100891493, another* ***MAOB*** *LOC115920263, family of* ***ZNFX1*** *LOC115920262, LOC753001, LOC105443121, 7* genes | ***NXPE3*** *LOC105444087, unknown LOC115920264, LOC577525, I****MPA1*** *LOC100891888* |
|  | *LOC115920226* | ***MRPL3*** *LOC756465, unknown LOC100887836,* ***KYAT4*** *LOC592180* | ***GSTO****1 LOC587859,* ***ACSF3*** *LOC579933,* ***TRIQK***  *LOC105440874* |
| ***H. pulcherimmus*** | ***  *HPU_03731*  *HPU_03729* | End of scaffold | ***Dachs,*** *HPU_03730, Unknown,* ***MRPL3****L* |
|  | *HPU_16863 HPU_16865 HPU_16868* | ***Zfp457****,* ***CCDC85****, unknown* | ***TpaseL12, gene 24578, SNED1*** |
| *L. variegatus* | *RAG1L* on chromosome 2 | ***CARF****? LOC121409187,* ***TBL2*** *LOC121409184,* ***SRCR1*** *LOC121409183* | ***Tigger*** *LOC121406973,* ***PPP4C*** *LOC121409190,* ***FLOT2*** *LOC121409191* |
| ***P. lividus*** | ***  *Pliv24208 Pliv25600* | ***HARBI1,*** *Orct****, transposable element TU1 fragment of a H2B pseudogene*** | ***LMLN****,* ***Tuba1a, KMT5A-like, GTPB1*** |
|  | *Pliv05932*  *Pliv07007*  *Pliv05896*  *Pliv06957* | ***HE***, ***Orct1/2, TEL, UFL1*** | ***NXPE 1L, EIF2D*** |
|  | *Pliv29923* | ***SEPRL, SNX12, NXPE3L, HSP56*** | ***Dnmt1, RHOBTB1, HSP56, TAK1*** |
|  | *Pliv14367* | ***TEL, methyltransferase-like protein 6, HARBI1*** | ***LNX1, HE, WDR12*** |
|  | *Pliv04572* | ***Orct, transposable element TU1, fragment of a H2B pseudogene (2 copies)*** | ***BAK1, Tuba1a*** |

*Gene clusters. Upstream and downstream genes are relative to the whole cluster.

Abbreviations: ***ALP1*** – Antagonist of like heterochromatin protein 1, ***MAOB*** – Monoamine oxidase B, ***ZNFX1*** - Zinc Finger NFX1-Type Containing 1, ***NXPE3*** Neurexophilin And PC-Esterase Domain Family Member 3, ***LOC115920264*** – prostate and placenta-expressed protein, ***IMPA1*** – Inositol monophosphatase, ***MRPL3*** *–* Mitochondrial Ribosomal Protein L3, ***KYAT4***- kynyrenine aminotransferase IV, , mitochondrial, ***GSTO1*** – glutathione S-transferase omega 1, ***ACSF3*** – Malonate--CoA ligase ACSF3, mitochondrial, ***TRIQK*** – Triple QxxK/R motif-containing protein like, ***dachs*** – dachs lethal myosin , ***Zfp457*** – zinc finger protein 457, ***CCDC85A*** – coiled-coil domain containing 85A, TpaseL_12, ***gene 24578*** *-* DNA Damage Inducible 1, ***SNED1*** *–* Sushi, Nidogen And EGF-Like Domain-Containing Protein 1, ***CARF*** – calcium responsive transcription factor, ***TBL2*** – transducin beta like 2, ***SRCR1*** – Scavenger receptor cysteine-rich domain superfamily protein, ***LOC121406973*** – tigger transposase, ***PPP4C*** – Serine/threonine-protein phosphatase 4 catalytic subunit, ***FLOT2***  - Flotillin 2, ***Orct*** – organic cation transporter, ***LMLN –*** Leishmanolysine-like gene***, KMT5AL*** - Lysine Methyltransferase 5A, ***GTPB1 –*** GTP-binding protein 1, ***HE*** - hemagglutinin-esterase, ***Orct1/2*** – Organic cation transporter 2, ***TEL*** – translocation–Ets–leukemia, ***UFL1*** – Ufmylation transferase 1, ***EIF2D*** - Eukaryotic Translation Initiation Factor 2D, ***SNX12*** - Sorting nexin 12, ***HSP56 -*** Human selenium binding protein-1, ***DNMT1*** – DNA Methyltransferase 1, ***RHOBTB1*** – Rho Related BTB Domain Containing 1, ***TAK1*** – Transforming Growth Factor-Beta-Activated Kinase 1, ***LNX1*** - Ligand Of Numb-Protein X 1, ***WDR12*** – WD Repeat Domain 12, ***BAK1*** – BCL2 Antagonist/Killer 1, ***TUBA1A*** – Tubulin Alpha 1a.

**Table S3. Expression databases for *S. purpuratus* search**

| **bioproject/database** | **experiment** | **stage/tissue** | **SpRAG1L** | **SpRAGL** | **LOC105439619** | **LOC105444038** | **LOC115920171** | **LOC115920226** |
| --- | --- | --- | --- | --- | --- | --- | --- | --- |
| PRJNA627693  GSE149221 | Sp1 | Early sea urchin development | low | high | negl | negl | not found | not found |
|  | Sp2 |  | low | high | negl | negl | not found | not found |
|  | SP3 |  | high | high | negl | negl | not found | not found |
|  | SpEB |  | high | high | negl | negl | not found | not found |
|  | SpHB |  | high | high | negl | negl | not found | not found |
|  | SpMB |  | high | high | negl | negl | not found | not found |
|  | SpEG |  | high | high | negl | negl | not found | not found |
|  | SpLG |  | high | high | negl | negl | not found | not found |
| PRJNA554833  GSE134350 | DMSO | embryo | negl | low | negl | negl | not found | not found |
|  | DAPT |  | negl | low | negl | negl | not found | not found |
|  | W |  | negl | low | negl | negl | not found | not found |
| PRJNA81157 | adult testes | embryos, larvae, and adult tissues | negl | negl | negl | negl | negl | negl |
|  | adult radial nerve |  | negl | negl | negl | negl | negl | negl |
|  | adult ovary |  | negl | negl | negl | negl | negl | negl |
|  | adult gut |  | negl | negl | negl | negl | negl | negl |
|  | adult coelomocyte |  | negl | negl | negl | negl | negl | negl |
| PRJNA381801  GSE97448 | activated coelomocytes | coelomocyte/gut | negl | not found | not found | not found | not found | not found |
|  | activated gut |  | negl | not found | not found | not found | not found | not found |

**Table S4. Echinoidea *RAGL* Expression data from TSA databases and RNA-seq studies**

| **Sea urchin** | **Study** | **Tissue type** | **BioProject** | **TSA/SRA available** | **RAG1La expression** | **RAG2L expression** | **RAG1L type II expression** | **TSA reads match**  **(best)** |
| --- | --- | --- | --- | --- | --- | --- | --- | --- |
| ***Strongylocentrotus purpuratus*** | Transcriptomes of sea urchin(Strongylocentrotus purpuratus) embryos, larvae, and adult tissues were profiled using RNA-seq technologyDOI: 10.1016/j.ydbio.2013.11.019  DOI: [10.1101/gr.139170.112](https://doi.org/10.1101/gr.139170.112) | Testes,Ovary,Radial nerve, Coelomocytes, Gut | PRJNA81157 | GHFM01 | expressed | expressed |  |  |
|  | A single cell RNA-seq resource for early sea urchin development DOI: [10.1242/dev.191528](https://doi.org/10.1242/dev.191528) | Developmental stages (8 cell through late gastrula) | [PRJNA627693](https://www.ncbi.nlm.nih.gov/bioproject/PRJNA627693) |  | expressed | expressed | LOC105444038  LOC105439619 |  |
|  | Single cell RNA-seq in the sea urchin embryo show marked cell-type specificity in the Delta/Notch pathway  DOI: [10.1002/mrd.23181](https://doi.org/10.1002/mrd.23181) | embryos at early and late gastrula stages |  |  | expressed | expressed |  |  |
| ***Strongylocentrotus droebachiensis*** | Genomic Characterization of the Evolutionary Potential of the Sea Urchin *Strongylocentrotus droebachiensis* Facing Ocean Acidification DOI: [10.1093/gbe/evw272](https://doi.org/10.1093%2Fgbe%2Fevw272) | Larval stages | PRJNA301890 |  | expressed | not found |  |  |
| ***Lytechinus variegatus*** | Developmental single-cell transcriptomics in the *Lytechinus variegatus* sea urchin embryo DOI: [10.1242/dev.198614](https://doi.org/10.1242/dev.198614) | development tissues/cells |  |  | Low level or uncertain | not found |  |  |
| ***Psammechinus miliaris*** | Molecular mechanisms underpinning transgenerational plasticity in the green sea urchin *Psammechinus miliaris*  DOI[: 10.1038/s41598-018-37255-6](https://doi/) | larvae 2stages and adult |  | SRP102910 | expressed | not found |  |  |
| ***Echinometra sp.*** | Little evidence of adaptation potential to ocean acidification in sea urchins living in “Future Ocean” conditions at a CO2 vent DOI: [10.1002/ece3.5563](https://doi.org/10.1002%2Fece3.5563) | larvae | PRJEB30637 | HAMP01 | expressed | expressed |  | HAMP01001378.1  HAMP01001379.1 HAMP01003424.1 HAMP01004817.1 HAMP01010307.1 HAMP01010308.1 HAMP01015319.1 HAMP01015320.1 HAMP01015605.1 HAMP01015608.1 HAMP01015656.1 HAMP01017939.1 HAMP01020826.1 |
| ***Mesocentrotus franciscanus*** | Gene expression patterns of red sea urchins (*Mesocentrotus franciscanus*) exposed to different combinations of temperature and *p*CO2 during early development DOI:10.1186/s12864-020-07327-x | development | PRJNA637102 PRJNA531463 | GHJZ01 | expressed | expressed |  | GHJZ01082967.1  GHJZ01085012.1 GHJZ01093105.1 GHJZ01030926.1 GHJZ01029345.1 GHJZ01039794.1 GHJZ01023892.1 |
| ***Loxechinus albus*** | De novo Assembly and Analysis of Tissue-Specific Transcriptomes of the Edible Red Sea Urchin Loxechinus albus Using RNA-Seq DOI: [10.3390/biology10100995](https://doi.org/10.3390/biology10100995) | intestines, gonads, and coelomocytes | PRJNA475570 | GGVM01 | expressed | expressed |  | GGVM01013856.1 GGVM01040411.1 GGVM01013855.1 |
| ***Evechinus chloroticus*** | The transcriptome of the NZ endemic sea urchin Kina (Evechinus chloroticus) DOI:10.1186/1471-2164-15-45 | adult | PRJNA190637 | GAPB01 | expressed | expressed |  | GAPB01003278.1  GAPB01063365.1 GAPB01008000.1 GAPB01020750.1 GAPB01045749.1 |
| ***Arbacia punctulata*** | EchinoDB, an application for comparative transcriptomics of deeply-sampled clades of echinoderms DOI:10.1186/s12859-016-0883-2 | adult | PRJNA299547 | GECD01 | expressed | expressed |  | GECD01034179.1 GECD01048449.1 GECD01053611.1 |
| ***Echinarachnius parma***  ***Eucidaris tribuloides***  ***Sphaerechinus granularis*** | Adrian Reich; 2014-01-22  Phylogenetic analysis of Echinoderms : Unpublished – Reich,A., Wessel,G. | ovary | PRJNA236087 | GAVR01  GAZP01  GAVF01 | expressed | expressed |  | GAVR01012962.1 GAVR01070653.1 GAVR01070662.1 GAVR01070663.1 GAVR01070656.1  GAZP01042081.1 GAZP01018122.1  GAVF01032930.1 GAVF01032929.1 GAVF01009690.1 GAVF01032928.1 |
| ***P. lividus*** | Developmental time course of Paracentrotus lividus embryogenesis : Unpublished –Malik,A., Sher,N., ben Tabou de Leon,S. | developmental | PRJNA264358 | GCZS00000000.1 | Low level or uncertain | Low level or uncertain | expressed | GCZS01118861.1  GCZS01087287.1  GCZS01087287.1  GCZS01132703.1  GCZS01085026.1 |
|  | Translatome analysis at fertilization in Paracentrotus lividus : Unpublished –  Morales,J., Chasse,H., Boulben,S., Aubert,J., Le Corguille,G., Corre,E., Cormier,P. | developmental | PRJNA288758 | GEDS00000000.1 |  |  | Low level or uncertain | GEDS01007385.1 |
|  | Developmental and housekeeping genes evolve under different constraints and show different positions of the phylotypic stage during sea urchin embryogenesis : Unpublished Ben-Tabou de-Leon,S. | developmental | PRJNA376650 | GFRN00000000.1 | Low level or uncertain | Low level or uncertain | expressed | GFRN01302615.1  GFRN01004376.1  GFRN01034014.1  GFRN01023862.1  GFRN01023864.1  GFRN01023865.1  GFRN01023866.1 |
|  | Integrative transcriptome and proteome analysis of the tube foot and adhesive secretions of the sea urchin Paracentrotus lividus : Unpublished Pjeta,R., Lindner,H., Kremser,L., Salvenmoser,W., Sobral,D., Ladurner,P., Santos,R. | tubefeet | PRJNA602659 | GIIR00000000.1 |  |  | expressed | GIIR01004244.1  GIIR01004245.1  GIIR01064023.1  GIIR01064024.1 |
|  | Genome-wide identification of genes activated by VEGF signaling in the sea urchin embryo : Unpublished Roopin,M., Ben-Tabou de-Leon,S | developmental | PRJEB10269 | HACU00000000.1 |  |  | expressed | HACU01282350.1  HACU01282351.1  HACU01282355.1  HACU01299379.1  HACU01299380.1  HACU01335064.1  HACU01479049.1 |

**Table S5 Sequences used in the article**

| **Gene mentioned in the article** | **Accession gene** | **Accession protein** | **Genomic location** |
| --- | --- | --- | --- |
| ***SpRAG1l typeI*** | **SpRAG1L*** | AAZ23546.1 | NW_022145614.1 2965149-2981235 |
| ***SpRAG1L3 type II*** | LOC105439619* | XP 011667102.2 | NW_022145599.1 30096128-30099255 |
| ***SpRAG1L 2 type II*** | SPU_015136.1* | Sp-Rag1l4 | KU668450.1 61407-64279 |
| ***SpRAG1L1 type II*** | LOC115920171* | XP 030831063.1 | NW_022145614.1 17008778-17010836 |
| ***SpRAG1L2 type II*** | LOC115920226* | XP 030831396.1 | NW_022145614.1 1267988-1269010 |
| ***PlRAG1a*** | Pliv25095.1** | Pliv25095.1 | CALNVZ010002100.1 25271589-25280514 |
| ***PlRAG1b*** | Pliv07077.1** | Pliv07077.1 | CALNVZ010003430.1 4057213-4065115 |
| ***PlRAG2L*** | Pliv25741.1** | Pliv25741.1 | CALNVZ010002100.1 25254955-25266487 |
| ***PlRAG1 1 type II*** | Pliv04572.1** | Pliv04572.1 | CALNVZ010000174.1 34432621-34438671 |
| ***PlRAG12 type II*** | Pliv06957.1** | Pliv06957.1 | CALNVZ010003430.1 4067542 - 4072001 |
| ***HpRAG1 type I*** | HPU_07483*** | HPU_07483 | BEXV01002133.1 6524-8912 |
| ***HpRAG1 1 type I*** | HPU 11753*** | HPU 11753 | BEXV01003119.1 26516-29835 |
| ***HpRAG1 2 type II*** | HPU 03730*** | HPU 03730 | BEXV01001680.1 4075-4800 |
| *PlArtemisL* | Pliv23693.1** | Pliv23693.1 | CALNVZ010002100.1 65779878-65791004 |
| *PlTdTL* | Pliv29804.1** | Pliv29804.1 | CALNVZ010003432.1 10833065-10843042 |

Gene accession numbers are given in: *NCBI database,

** *P.lividus* database <http://octopus.obs-vlfr.fr/blast/oursin/blast_oursin.php>

*** *H. pulcherrimus* database <https://cell-innovation.nig.ac.jp/cgi-bin/Hpul_public/Hpul_annot_home.cgi>
